# Some Points in the Diagnosis and Treatment of Accidental Hemorrhage

**Published:** 1907-03

**Authors:** 


### SOME POINTS IN THE DIAGNOSIS AND TREATMENT OF ACCIDENTAL HEMORRHAGE.

Wright (American Journal of Obstetrics and Diseases of Women and Children, November, 1906) holds that the diagnosis in many cases of concealed accidental hemorrhage is generally difficult and sometimes impossible before delivery, since the important symptoms —i. e., anemia and distention of the uterus—are not present in such cases. The immediate symptoms are usually embraced in the term shock from traumatism, and not collapse from loss of blood.

When the hemorrhage is both internal and external the diagnosis is most readily made, but the effect of the blood within the uterus is often difficult to ascertain. Even in such cases the shock from traumatism is often the predominating element, though exceptionally collapse from loss of blood, whether retained within the uterus or flowing externally, may be an important factor. In all cases in which shock is the main condition or the predominating element, the treatment of shock is the one of greatest urgency. In most cases of combined internal and external hemorrhage the introduction of the vaginal plug, with the application of an abdominal binder, appears to be a very safe and effectual plan of treatment.

In a small proportion of cases, especially during labor, puncture of the membrane is beneficial. Any form of forced delivery which includes rapid dilatation of a rigid cervix is unjustifiable. The best operative procedure would appear to be some form of vaginal section, but the field for this is limited and not accurately defined.

